# A granular mechanics model study of the influence of non-spherical shape on aggregate collisions

**DOI:** 10.1038/s41598-023-29247-y

**Published:** 2023-02-06

**Authors:** Rahul Bandyopadhyay, Herbert M. Urbassek

**Affiliations:** Physics Department and Research Center OPTIMAS, University Kaiserslautern, Erwin-Schrödinger-Straße, 67663 Kaiserslautern, Germany

**Keywords:** Astronomy and planetary science, Materials science

## Abstract

Collisions between granular aggregates influence the size distribution of dust clouds. Granular aggregates may possess non-spherical shapes as a result of, for instance, previous collision processes. Here, we study aggregate collisions using a granular mechanics simulation code. Collisions between spherical aggregates are compared to collisions of ellipsoidal aggregates of equal mass. As the most prominent result, we find that the growth velocity, i.e., the velocity above which the post-collision aggregates are smaller than before collision, is generally reduced for ellipsoidal aggregates. The reason hereto lies in the less compact structure of ellipsoids which allows for a larger degree of fragmentation in a ‘rim peel-off’ mechanism. On the other hand, relative fragment distributions are only little influenced by aggregate shape.

## Introduction

Collisions between dust particles are ubiquitous in the space environment and can be found in, e.g., protoplanetary disks^1^ and debris disks^2^. Such dust particles are aggregates of individual grains. Since the aggregation process can be assumed to proceed isotropically, studies of dust aggregates usually assume an approximately spherical shape of the aggregates^3–8^. In the past, a considerable number of both experimental^7,9,10^ and simulational^11–18^ studies of aggregate collisions have been performed.

In such collisions, major interest lies in the determination of whether the colliding aggregates merge to form a larger aggregate, or fragment into smaller pieces. The former scenario means ‘growth’ and is the first step in the route how larger aggregates and finally planetesimals form in protoplanetary disks^19,20^. Monte Carlo codes^21,22^ that simulate the temporal evolution of such disks therefore take as their input collision results from experiment or granular simulations, in which the collisional outcomes (growth or erosion) are tabulated as a function of collisional velocity, aggregate porosity, and possibly other parameters.

However, after the collisional merging of aggregates, the resulting collision product will no longer be of a spherical shape. While the actual shape of the merged aggregate—and also of the fragments in the case of eroding collisions—may be very varied, the simplest model may consist in ascribing the merged aggregate the form of a prolate axisymmetric ellipsoid, in which the symmetry axis corresponds to the collision axis of the generating collision.

In the present work, we study collisions of such ellipsoidal aggregates with spherical aggregates and among themselves. Rather than attempting a systematic approach, we wish to explore which features of the collisions between spherical aggregates are (strongly) affected by the aggregate shape, and which not. Therefore, we only study a single aspect ratio of the ellipsoids of 2:1, motivated by the above mentioned formation by a merging collision. We explore collisions of ellipsoidal aggregates with spherical ones of the same mass, and finally also collisions between ellipsoidal aggregates and compare them with the reference case of collisions between spherical particles. Only central collisions are studied since the parameter space for describing oblique collisions systematically is too vast, in particular for collisions between ellipsoids.

## Methods

**Table 1:** Nomenclature of collision set-ups for *sph-ell* and *ell-ell* collisions.

Set-up nomenclature	Collision type	Orientation of the ellipsoidal aggregate(s)
${\hat{\varvec{c}}}\perp {\varvec{v}}$	*sph-ell*	$\hat{\varvec{c}}$ is perpendicular to $\varvec{v}$
${\hat{\varvec{c}}}45^{\circ }{\varvec{v}}$	*sph-ell*	$\hat{\varvec{c}}$ is at an angle of $45^{\circ }$ to $\varvec{v}$
${\hat{\varvec{c}}}\parallel {\varvec{v}}$	*sph-ell*	$\hat{\varvec{c}}$ is parallel to $\varvec{v}$
${\hat{\varvec{c}}_{1}}\parallel {\hat{\varvec{c}}_{2}}\parallel {\varvec{v}}$	*ell-ell*	$\hat{\varvec{c}}_{1}$ and $\hat{\varvec{c}}_{2}$ are parallel to each other, and also parallel to $\varvec{v}$
$({\hat{\varvec{c}}_{1}}\parallel {\hat{\varvec{c}}_{2}})\perp {\varvec{v}}$	*ell-ell*	$\hat{\varvec{c}}_{1}$ and $\hat{\varvec{c}}_{2}$ are parallel to each other, but perpendicular to $\varvec{v}$
$({\hat{\varvec{c}}_{1}}\parallel {\varvec{v}})\perp {\hat{\varvec{c}}_{2}}$	*ell-ell*	$\hat{\varvec{c}}_{1}$ is parallel to $\varvec{v}$, but perpendicular to $\hat{\varvec{c}}_{2}$
${\hat{\varvec{c}}_{1}}\perp {\hat{\varvec{c}}_{2}}\perp {\varvec{v}}$	*ell-ell*	$\hat{\varvec{c}}_{1}$, $\hat{\varvec{c}}_{2}$ and $\varvec{v}$ are all perpendicular to each other

### Granular mechanics model

The granular mechanics model used in simulating aggregate collisions is based on the fundamental studies by Tielens et al.^11,24–26^. The code has been described in detail in^27^, such that only a few major ingredients are mentioned in the following.

The code describes aggregates composed of monodisperse spherical grains of radius *r*. Grains interact when the distance of their centers is smaller than 2*r*. The interaction forces can be split into normal forces acting along the line connecting the centers of the interacting grains and tangential forces which lead to torques on the grains. The normal force includes elastic repulsion following Hertz’ law, a viscous drag proportional to the normal component, $v_n$ of their relative velocity, and an adhesive force which is modeled to be constant for simplicity. Using the grain overlap $\delta$, the specific surface energy $\gamma$, Young’s modulus *Y* and the Poisson number $\nu$ of the grain material as well as the dissipation constant *A*, the normal force is given by

1 $$\begin{aligned} F_n = -2\pi \gamma r + \frac{2}{3}\frac{Y}{1-\nu ^2}\sqrt{\frac{r}{2}} \delta ^{3/2} - A\sqrt{\frac{\delta r}{2}} v_n. \end{aligned}$$

The tangential contributions to the force and torques include rolling, sliding and twisting motion of the grains and are given in detail in^27^.

### Aggregate configuration

Aggregates are built from monodisperse silica grains. These grains have a radius of $r=0.76$ μm and a mass of $m= 3.68 \times 10^{-15}$ kg, corresponding to a mass density of $\rho =2000$ kg/m$^3$. Their material properties are described by $\gamma =0.025$ J/m$^2$, $Y=54$ GPa, $\nu =0.17$, $\rho =2000$ kg/m$^3$^27^. The dissipation constant related to the viscous friction of the normal motion is chosen as $A=0.5$ ns^27^. The equilibrium overlap, i.e., the overlap at which the normal force vanishes, can be calculated from Eq. (1) as $\delta _{\textrm{equ}}= 3.01$ Å.

Aggregates are characterized by the number of grains, *N*, they contain and the filling factor, $\Phi$. It is defined as the ratio of the total volume of the grains in the aggregate, $N(4\pi /3)r^3$, and the volume of the aggregate, $V_{\textrm{agg}}$. In the present work, we use $N=20{,}000$ and $\Phi =0.24$.

Since the seminal work of Dominik and Tielens^11^, it is assumed that the outcome of aggregate collisions mainly depends on the ratio of the collision energy, $E= N (m/4) v^2$, to the total energy needed to break grain contacts, $E_{\textrm{sep}} \approx N E_{\textrm{fr}}$, where $E_{\textrm{fr}}$ is the energy needed to break a single contact, and hence only on the collision velocity. Indeed, simulations of aggregate collisions demonstrated that relevant quantities characterizing the collision outcome depend on collision velocity rather than energy and are quite insensitive to aggregate size^14,15,18^. For this reason, our simulations with a single aggregate size, $N=20{,}000$, should be expected to be valid for larger aggregates as well, if the results are scaled to collision velocity rather than collision energy.

On the other hand, collision outcomes do depend on aggregate porosity; recent studies showed that the fragmentation probability increases for aggregates of smaller filling factors^28,29^, even though the dependence on $\Phi$ is weak. Thus our results for $\Phi =0.24$ should not carelessly be generalized to other filling factors.

The radius *R* of spherical aggregates is thus given by

2 $$\begin{aligned} R=\left( \frac{N}{\Phi }\right) ^{1/3}r \end{aligned}$$

and amounts to $R=33.2$ μm.

We assume ellipsoidal aggregates to be axisymmetric prolate ellipsoids with semi-major axis *c* and semi-minor axis $a=c/2$. Hence,

3 $$\begin{aligned} c=\left( \frac{4N}{\Phi }\right) ^{1/3}r \end{aligned}$$

amounting to $c=52.7$ μm.

Spherical and ellipsoidal aggregates are constructed by a random process. As the geometrical shape and the number of grains to put into the shape are known, we start by putting a grain at the center of the shape. Then we iterate the following algorithm until *N* grains are placed: we attach a new grain to a randomly chosen grain in random direction. This attachment occurs at a distance of $2r-\delta _{\textrm{equ}}$, where $\delta _{\textrm{equ}}$ is the equilibrium overlap of two grains. If the newly added grain is not entirely within the prescribed aggregate shape or if its distance to another grain is smaller than $2r-\delta _{\textrm{equ}}$, it is deleted, otherwise it is kept. After aggregate construction, we relax the aggregate by running a short simulation to reduce any tangential and normal forces that might have built up during the construction process.

### Simulation set-up

We run simulations of collisions between two aggregates. The collision events can be categorized into three main types: (i) both the aggregates are spherical: *sph-sph*, (ii) one of the aggregates is spherical and the other one is ellipsoidal: *sph-ell*, and (iii) both the aggregates are ellipsoidal: *ell-ell*. The collisions events in our simulations all run in their centre-of-mass (CM) coordinate system. The aggregates are set to approach towards each other along the line joining their centers with velocities *v*/2, such that the relative velocity measures *v* and the impact parameter of the collision is zero.

**Figure 1:**
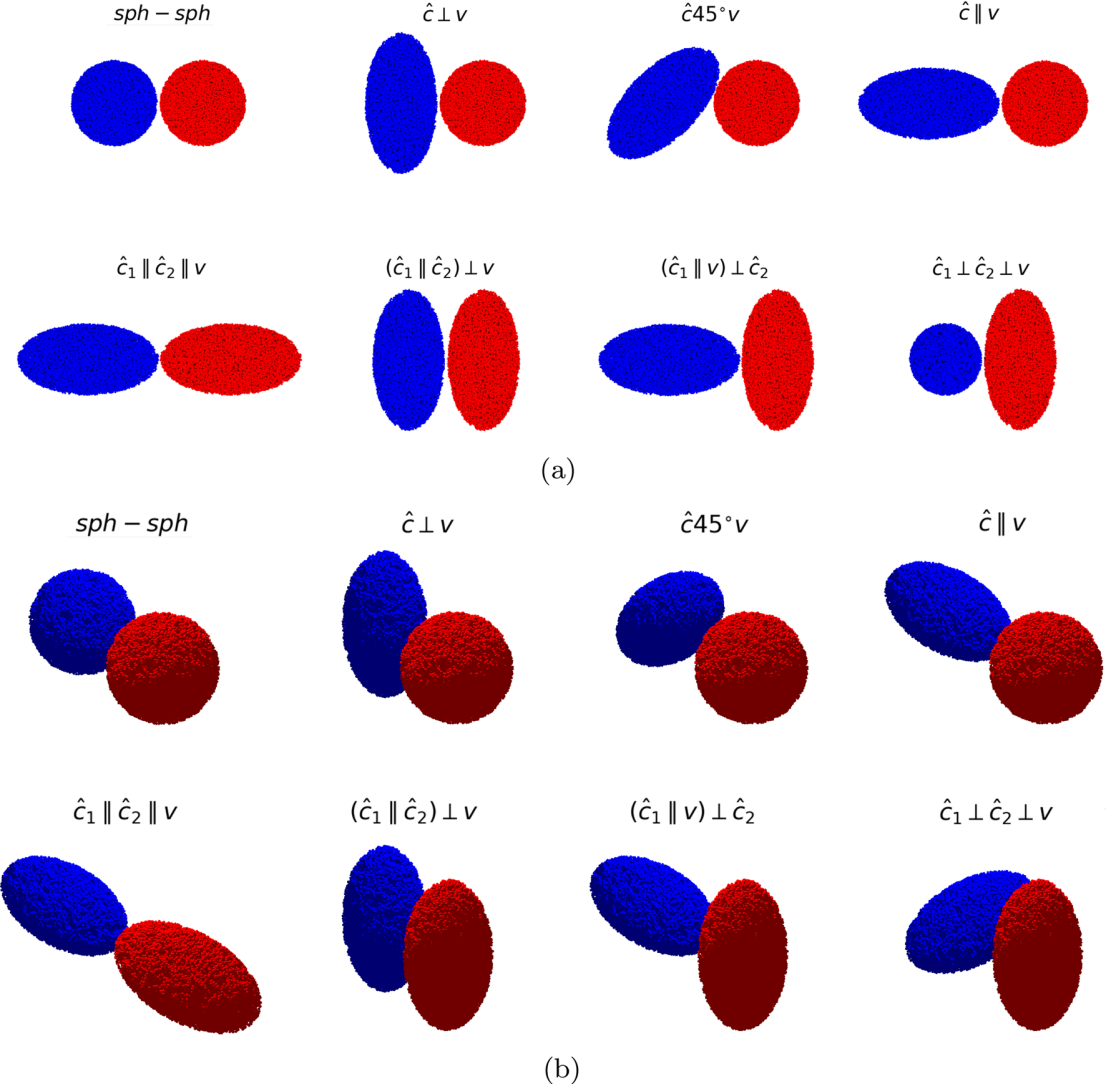
Set-ups of all collision events studied in this work. (**a**) Plane view; the collision velocity is in the horizontal direction. (**b**) 3D view, in which the viewing direction makes an angle of $45^\circ$ with all axes. A light source is placed on the top of each setup to illuminate the aggregates for emphasizing their 3D shape and orientation. Figure created using POV-Ray^23^.

Figure 1 shows the initial set-ups of the collision events simulated in this work. The unit vector along the major axis of the ellipsoids is denoted by $\hat{\varvec{c}}$; for *ell-ell* events, the two ellipsoid orientations are denoted by $\hat{\varvec{c}}_{1}$ and $\hat{\varvec{c}}_{2}$. The initial set-ups are obtained by varying the orientation $\hat{\varvec{c}}$, $\hat{\varvec{c}}_{1}$ and $\hat{\varvec{c}}_{2}$ with respect to the velocity, $\varvec{v}$. The set-ups for *sph-ell* and *ell-ell* collision events are explained in Table 1 with the nomenclature used in this paper.

Note that only high-symmetry geometries are studied. The only exception is ${\hat{\varvec{c}}}45^{\circ }{\varvec{v}}$; along with ${\hat{\varvec{c}}}\perp {\varvec{v}}$ and ${\hat{\varvec{c}}}\parallel {\varvec{v}}$, this configuration completes the overview of central *sph-ell* collisions. The analogous overview over all central *ell-ell* collisions would be computationally too costly, since in general the axes $\hat{\varvec{c}}_{1}$ and $\hat{\varvec{c}}_{2}$ may have an arbitrary orientation with respect to $\varvec{v}$, giving rise to 3 angle parameters to sample.

Each of the collision set-ups shown in Fig. 1 are simulated for six velocities *v*: 2.5, 3.75, 5, 6.25, 7.5, 8.75, 10, 12.5, 15 m/s. Each simulation runs for 200 μs in steps of $\Delta t=50$ ps.

### Identification of fragments

As a result of a collision event, the aggregates may merge and/or fragment. We quantify the size of the fragments by the number of grains contained in that fragment. The algorithm by Stoddard^30^ is used to determine the aggregate sizes.

## Results and discussion

### Overview over collision outcomes

**Figure 2:**
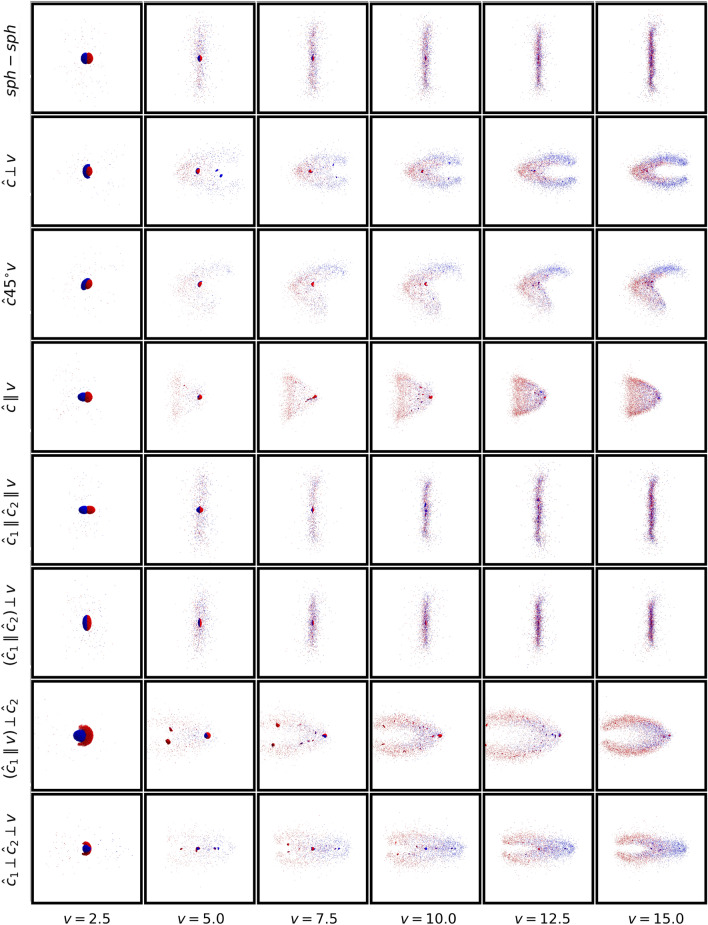
Tableau of the final state after the collision of two aggregates colliding with velocity *v* (in units of m/s) and the collision geometries, cf. Table 1. Size of the space plotted in the subpanels changes with collision velocity. For the sake of a clear visualization of the monomers and small fragments, the grain size is appropriately scaled with the subpanel boxes. Grains are colored according to their initial aggregate affiliation. Figure created using POV-Ray^23^.

Figure 2 displays snapshots at the end of the simulation and thus gives an overview over the collision outcomes in its dependence on collision velocity and geometry. At the smallest velocity, $v=2.5$ m/s, the collision always generates a fused aggregate with only little grain ejection. At higher velocities, fragmentation processes become dominant; in these, a multitude of monomers and small fragments are ejected. However, a few examples also show up, where several large fragments form in the cloud of grains; this occurs for $v=5$ m/s for the $({\hat{\varvec{c}}_{1}}\parallel {\varvec{v}})\perp {\hat{\varvec{c}}_{2}}$ geometry, for instance, but also at higher velocities, for example for the ${\hat{\varvec{c}}_{1}}\parallel {\hat{\varvec{c}}_{2}}\parallel {\varvec{v}}$ geometry at 10 m/s. A quantification of the fragment distribution will be discussed later. We note that for central collisions, fusion and fragmentation are the only outcomes found in granular simulations; for oblique collisions, also sliding (also denoted by shearing or bouncing) collisions may occur^14,15^.

While the forms of the $v=2.5$ m/s merged clusters appear obvious from the collision geometries, Fig. 1, the shape of the fragment cloud developing at higher velocities becomes more complex. For the highly symmetrical cases *sph-sph*, ${\hat{\varvec{c}}_{1}}\parallel {\hat{\varvec{c}}_{2}}\parallel {\varvec{v}}$ and $({\hat{\varvec{c}}_{1}}\parallel {\hat{\varvec{c}}_{2}})\perp {\varvec{v}}$, the main fragment ejection occurs in the collision plane, i.e., the plane perpendicular to $\varvec{v}$ through the CM. In the other cases, the parts of the aggregate that lie farthest from the collision axis are stripped off. We will denote this mechanism as ‘rim peel-off’ in later discussions. It is possible in *sph-ell* collisions since the semi-major ellipsoid axis, $c=52.7$ μm, strongly surpasses the sphere radius $R=33.2$ μm; in *ell-ell* collisions such stripping processes occur whenever ${\hat{\varvec{c}}_{1}}$ and ${\hat{\varvec{c}}_{1}}$ are not aligned. In all cases but the ${\hat{\varvec{c}}}45^{\circ }{\varvec{v}}$ geometry, symmetric fragment patterns appear in Fig. 2, however, these will not always be axisymmetric. Finally, we emphasize that the fate of the grains in the two colliding aggregates may be strongly different, as indicated by the blue and red colors in Fig. 2. Take as an example the ${\hat{\varvec{c}}}45^{\circ }{\varvec{v}}$ geometry of the *sph-ell* collision; at high velocities, material from the ellipsoid rims will be stripped off and travel forward essentially undisturbed, while the material of the sphere close to collision axis is stopped. Since the semi-minor ellipsoid axis is only $a=26.3$ μm and thus smaller than the sphere radius, sphere grains originating from the sphere rim in the direction perpendicular to the plane plotted in Fig. 2 are stripped off the aggregate and can move towards the left side.

**Figure 3:**
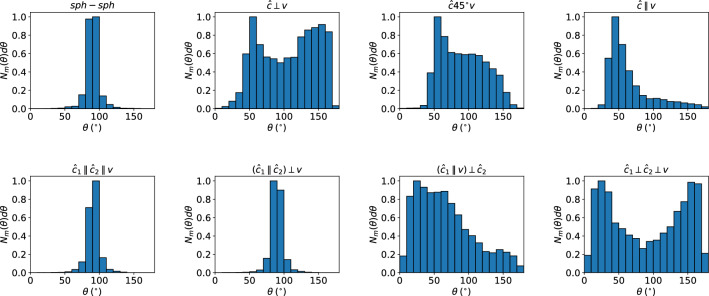
Angular distribution of monomers, $N_m(\theta )$, for collision velocity of $v=15$ m/s and the collision geometries indicated, cf. Table 1. Data are normalized to their peak values.

For further quantitative discussion of the collision outcome, we discuss the angular distribution of the ejecta in Fig. 3. For simplicity, we focus on the spherical polar angle $\theta$ of the ejecta with respect to the velocity vector, $\varvec{v}$. $\theta =0^{\circ }$ denotes motion to the left in Fig. 2, and $\theta =180^{\circ }$ motion to the right. We only determine the angular distribution of monomers, since these constitute the majority of fragments.

Figure 3 shows the focused ejection within the collision plane for the highly symmetrical cases *sph-sph*, ${\hat{\varvec{c}}_{1}}\parallel {\hat{\varvec{c}}_{2}}\parallel {\varvec{v}}$ and $({\hat{\varvec{c}}_{1}}\parallel {\hat{\varvec{c}}_{2}})\perp {\varvec{v}}$. Strong forward and backward ejection is found for the crossed-ellipsoid geometry ${\hat{\varvec{c}}_{1}}\perp {\hat{\varvec{c}}_{2}}\perp {\varvec{v}}$. The other cases feature forward-backward asymmetry which has varied origins: in ${\hat{\varvec{c}}}45^{\circ }{\varvec{v}}$ and ${\hat{\varvec{c}}}\parallel {\varvec{v}}$, the shape of the colliding aggregates makes the difference, in ${\hat{\varvec{c}}}45^{\circ }{\varvec{v}}$ the additional tilt angle of the ellipsoid plays a role. Finally, in $({\hat{\varvec{c}}_{1}}\parallel {\varvec{v}})\perp {\hat{\varvec{c}}_{2}}$ the orientation of the two ellipsoids with respect to the collision axis is decisive for the emission pattern; the ellipsoid with ${\hat{\varvec{c}}}\parallel {\varvec{v}}$ has difficulties in penetrating the core of the second ellipsoid such that the majority of the ejecta lie on the left-hand side, $\theta < 90^{\circ }$.

### Critical velocities

The question of whether a collision of two aggregates leads to their fusion or rather to fragmentation is often of prime interest in collision studies, since it decides on whether aggregates grow or erode by collisions. This question can be decided with the help of the number of grains in the largest post-collisional aggregate, $N_1$. By normalizing to the number of grains initially present in one aggregate, we thus define the *growth ratio*

4 $$\begin{aligned} n_{\textrm{gr}}= \frac{N_1}{N} . \end{aligned}$$

We will denote collisions with $n_{\textrm{gr}}> 1.5$ as fusion outcomes, even though there certainly exists an arbitrariness in which limiting value to use (instead of 1.5). In the literature, often $n_{\textrm{gr}}> 1$ is characterized as the ‘growth regime’; the quantity $n_{\textrm{gr}}-1$ has been denoted the ‘erosion efficiency’^29,31,32^. Growth is thus a weaker concept than fusion.

**Figure 4:**
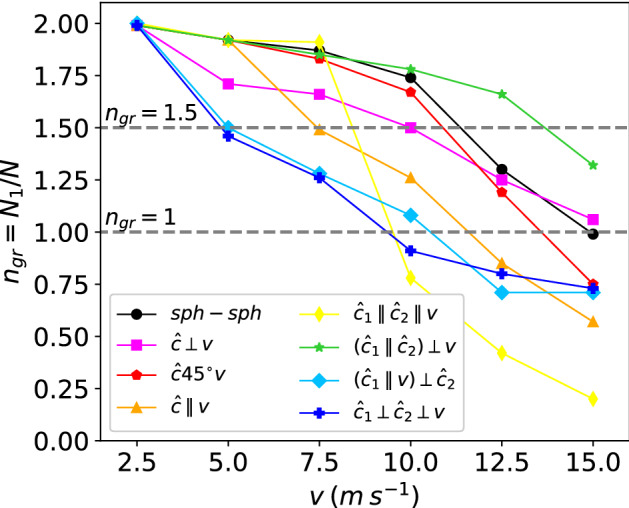
Dependence of the growth ratio, $n_{\textrm{gr}}$, Eq. (4), on collision velocity, *v*. The dotted lines delineate the growth regime, $n_{\textrm{gr}}>1$, and the fusion regime, $n_{\textrm{gr}}> 1.5$.

Figure 4 shows the dependence of the growth ratio with velocity for the collision geometries investigated. Not surprisingly, $n_{\textrm{gr}}$ is monotonically decreasing with velocity as fragmentation processes gain in importance. The velocities, $v_{\textrm{fu}}$, at which $n_{\textrm{gr}}$ crosses the value 1.5 may be taken to mark the end of the fusion regime; where it passes the value of 1, $v_{\textrm{gr}}$, as the end of the growth regime.

Interestingly, the case of *sph-sph* collisions shows quite large values of $n_{\textrm{gr}}$ compared to the other geometries. This quantifies our above discussion that fragmentation is more abundant in *sph-ell* and *ell-ell* collisions than in *sph-sph* collisions. The compact shape of spherical aggregates minimizes fragmentation; the grains in the rim of the ellipsoids far away from the collision axis are prone to lead to monomer (and small fragment) ejection, thus reducing the growth ratio $n_{\textrm{gr}}$. Note that aggregate shape has a strong effect here: the fusion velocity, $v_{\textrm{fu}}$, may be reduced from the value of around 11.5 m/s for *sph-sph* collisions to only 5 m/s for unfavorable geometries. The decrease for the growth velocity is smaller; from $v_{\textrm{gr}}=15$ m/s for the *sph-sph* case to only 10 m/s in other geometries.

One might have expected that the ${\hat{\varvec{c}}}45^{\circ }{\varvec{v}}$ collision interpolates between the ${\hat{\varvec{c}}}\perp {\varvec{v}}$ and ${\hat{\varvec{c}}}\parallel {\varvec{v}}$ geometries; Fig. 4 shows that this is not the case. In the ${\hat{\varvec{c}}}\perp {\varvec{v}}$ geometry, the ellipsoid rims close to the major axis do not interact with the colliding sphere and are therefore prone to fragmentation; in the ${\hat{\varvec{c}}}45^{\circ }{\varvec{v}}$ geometry, because of the tilt angle, these far-lying ellipsoid rims are brought closer to the collision axis and thus contribute less to fragmentation. In the ${\hat{\varvec{c}}}\parallel {\varvec{v}}$ geometry, on the other hand, parts of the sphere are far from the interaction region; this geometry fragments most among the *sph-ell* collisions. These results thus demonstrate that aggregate fragmentation is minimum if the aggregate cross sections perpendicular to the collision axis show maximum overlap.

We note that for some geometries, such as ${\hat{\varvec{c}}}\perp {\varvec{v}}$ and in particular ${\hat{\varvec{c}}}45^{\circ }{\varvec{v}}$, the geometry effect is only small; for $({\hat{\varvec{c}}_{1}}\parallel {\hat{\varvec{c}}_{2}})\perp {\varvec{v}}$, fragmentation is even less than for *sph-sph* collisions. Here, the ellipsoid axes are aligned such that the ellipsoids collide with their maximum cross section facing each other; this leads to efficient stopping of the aggregate motion and to easy energy dissipation. In contrast, when the ellipsoids collide with their minimum cross sections facing each other, ${\hat{\varvec{c}}_{1}}\parallel {\hat{\varvec{c}}_{2}}\parallel {\varvec{v}}$, strong fragmentation sets in beyond 7.5 m/s.

We conclude that deviations from the spherical shape are prone to induce more fragmentation and diminish the fusion and growth velocities. This behavior is caused by aggregate parts far from the collision axis that are stripped off during the collision (‘rim peel-off’). Only for specially selected cases, where the aggregates impact on each other with maximum cross section, can fragmentation be reduced below the value of *sph-sph* collisions.

### Fragment size distribution

**Figure 5:**
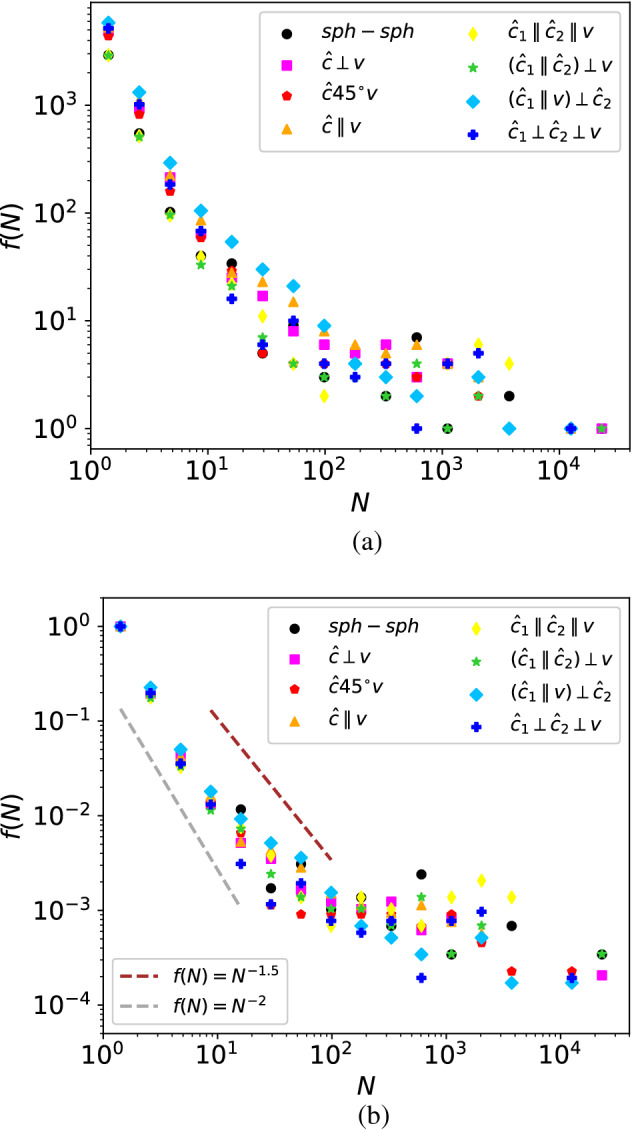
(**a**) Size distribution of fragments, *f*(*N*), for collision velocity of $v=15$ m/s and the collision geometries indicated, cf. Table 1. (**b**) Data are normalized to $f(1)=1$. Dashed lines show power-law behavior, Eq. (5), with $\tau =2$ and 1.5 for orientation.

In cases of high-speed collisions, where many fragments are formed, the size distribution of fragments, *f*(*N*), is relevant. Figure 5a displays this distribution for all geometries investigated for the highest velocity investigated, 15 m/s. The data emphasize our above discussion of the growth ratios. For instance, in the $({\hat{\varvec{c}}_{1}}\parallel {\hat{\varvec{c}}_{2}})\perp {\varvec{v}}$ geometry with its high growth velocity, the number of fragments with sizes $\lesssim 10^3$ is minimum, corresponding to little fragmentation. On the other hand, for the $({\hat{\varvec{c}}_{1}}\parallel {\varvec{v}})\perp {\hat{\varvec{c}}_{2}}$ geometry, the number of small fragments ($N \lesssim 10^2$) is maximum. Also note that the largest fragments, $N \gtrsim 10^4$, correspond to the remnants of the original colliding aggregates.

The fall-off of the fragment-size distribution has often been described with a power-law^13–15,21^

5 $$\begin{aligned} f(N) \propto N^{-\tau } . \end{aligned}$$

The curvature of the distribution in the double-logarithmic plot, Fig. 5, demonstrates that such a fit is possible only in a finite fragment-size interval. Our data allow a fit with $\tau =2$ in the low fragment-size range, $N \le 10$, and $\tau =1.5$ for larger *N*, $10 \le N \le 100$, in qualitative agreement with previous studies^13–15^. Remarkably, the power-law exponent does not appear to depend sensitively on the collision geometry.

**Figure 6:**
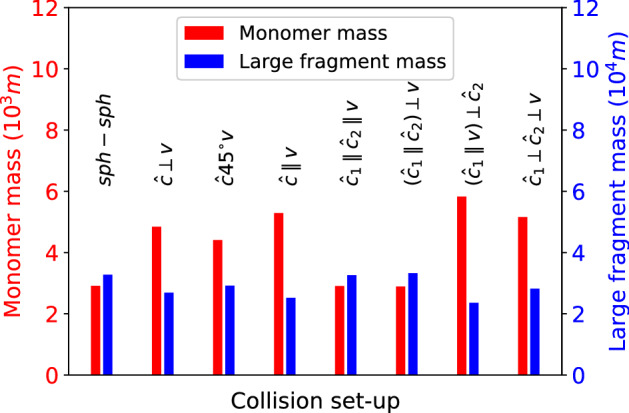
Dependence of the total mass of ejected monomers, $N_m$, and the total mass of large fragments with sizes $N\ge 400$ on the collision geometry, cf. Table 1.

Figure 6 compares the numbers of monomers in the various collision geometries investigated. As discussed above, the dependence on geometry amounts to a factor of 2 at most. Most monomers are obtained for the $({\hat{\varvec{c}}_{1}}\parallel {\varvec{v}})\perp {\hat{\varvec{c}}_{2}}$ geometry—where up to $N = 10^2$ the number of fragments is maximum. The smallest number of monomers is obtained for the ${\hat{\varvec{c}}_{1}}\parallel {\hat{\varvec{c}}_{2}}\parallel {\varvec{v}}$ and the $({\hat{\varvec{c}}_{1}}\parallel {\hat{\varvec{c}}_{2}})\perp {\varvec{v}}$ geometries, where the aligned ellipsoid axes do not allow for fragment formation by the ‘rim peel-off’ effect discussed above.

Besides the grain-size distribution, it is also relevant to discuss the distribution of aggregate masses in the fragments. The mass of a fragment containing *N* grains amounts to *mN*. While for monomers, the masses do not give any further information, the mass contained in large fragments is substantially larger than the $N^{-\tau }$ decay of the size distribution, Eq. (5), suggests. In Fig. 6, we also show the total mass of ‘large’ fragments, defined as fragments with $N \ge 400$, i.e., fragments that contain at least 1 per cent of the total mass of the system. In this mass regime, the size distribution, Fig. 5, is rather flat and shows strong fluctuations caused by the small numbers of fragments generated with these high masses. Figure 6 shows that the masses contained in these large clusters are more than an order of magnitude higher than the—more abundant—monomers. Large fragments are most dominant in the $({\hat{\varvec{c}}_{1}}\parallel {\varvec{v}})\perp {\hat{\varvec{c}}_{2}}$ and ${\hat{\varvec{c}}_{1}}\perp {\hat{\varvec{c}}_{2}}\perp {\varvec{v}}$ geometries; as Fig. 5 shows, in these cases the fragments with sizes in the range of hundreds to thousands dominate, but not the remnants of the colliding clusters with sizes $> 10^4$.

## Summary

We used granular mechanics simulations to study collisions between granular aggregates with the aim to explore which features are most pronouncedly affected by aggregate shape. We obtained the following findings for equal-mass aggregates colliding centrally.

1. Aggregate shape plays a major role in the onset of fragmentation processes. These can be conveniently quantified using the size of the largest post-collision aggregate, $N_1$. Collisions of compact aggregates (such as spheres) or collisions where aggregates face each other with a large cross section lead to large $N_1$.
2. Conversely, collisions of aggregates with incompatible facing cross-sections lead to an increase in fragmentation seen in the number of ejected monomers. This can be explained by the ‘rim peeling’ mechanism: Those aggregate parts that are distant from the collision axis and do not face the collision partner are stripped off during the collision.
3. This geometrical effect strongly influences the critical velocities for fusion and for growth. These velocities are needed in calculations of the collisional evolution of dust clouds. Our study thus emphasizes that aggregate shape may be factor that needs to be taken into account in such calculations.
4. Fragment-size distributions, on the other hand, are only little affected by aggregate shape.

The parameters describing the intergranular forces have been chosen in this study to describe the interaction between silica grains of radius $r=0.76$ μm, cf^27^. In particular, the dissipation constant *A* was chosen to fit available experimental data for the coefficient of restitution^27,33^. Similarly, the specific surface energy $\gamma$ is in agreement with available data for amorphous silica spheres as reviewed in^34^. Nevertheless, it is of interest to discuss which changes are induced in the collision dynamics of aggregates when these parameters are changed. This question was studied previously for simpler collision scenarios, viz., collisions between spherical aggregates or with a granular bed having a planar surface. It was found that a reduction of dissipation, *A*, (i) increases the coefficient of restitution between two grains^27^; (ii) in aggregate collisions, it increases fragmentation, since more kinetic energy remains available for grain bouncing and hence aggregate fragmentation^17^. The effects of changes in the surface energy—and hence the grain adhesion—on aggregate collisions has to our knowledge not been studied with comparable care. However, we mention that an increased $\gamma$ leads to a quicker stopping of the aggregates^35^ and a stronger material compaction^36^; hence it can be expected to lead to less fragmentation in aggregate collisions, even though this aspect does not seem to have been investigated systematically.

In future studies, it may be interesting (i) to provide more systematics to the collision outcomes as a function of the ellipsoid axis ratio; (ii) to study the outcomes of non-central collisions between spherical and aspherical particles; and (iii) to extend the aggregate shape to more general forms, in particular to non-axisymmetric shapes as they may originate from oblique collisions.

## Data Availability

All data used for this study are contained in this article.
